# Discovery of Novel Circulating Immune Complexes in Lupus Nephritis Using Immunoproteomics

**DOI:** 10.3389/fimmu.2022.850015

**Published:** 2022-03-24

**Authors:** Chenling Tang, Min Fang, Gongjun Tan, Shu Zhang, Bowen Yang, Yaxi Li, Ting Zhang, Ramesh Saxena, Chandra Mohan, Tianfu Wu

**Affiliations:** ^1^Department of Biomedical Engineering, University of Houston, Houston, TX, United States; ^2^Division of Nephrology, University of Texas, Southwestern Medical Center, Dallas, TX, United States

**Keywords:** immunoproteomics, immune complex (ICx), biomarkers, biomarker panel, systemic lupus erythematosus, lupus nephritis

## Abstract

**Objective:**

The goal is to discover novel circulating immune complexes (ICx) in the serum of lupus nephritis (LN) as potential biomarkers.

**Methods:**

Protein A/G magnetic beads or C1q-coated plates were used to capture ICx in the serum of LN, followed by the identification of immunoglobulin-binding proteins using liquid chromatography and tandem mass spectrometry (LC-MS/MS). Bioinformatic approaches and single-cell RNA sequencing (scRNA Seq) databases were used to select potential candidate ICx markers in LN. The selected ICx markers were further validated using ELISA.

**Results:**

A total of 300 immunoglobulin-binding proteins were discovered in the screening, among which 77 proteins were detectable only in LN samples. Bioinformatics-assisted selection allowed us to further identify 10 potential immunoglobulin-binding proteins, which form ICx as potential biomarkers in LN. In a validation cohort of 62 LN patients and 21 healthy controls (HC), we found that prolyl 3-hydroxylase 1 (P3H1), phosphatase and actin regulator 4 (PHACTR4), and regulator of G-protein signaling 12 (RGS12) ICx exhibited discriminative capability in distinguishing LN from HC, with an area under the curve (AUC) values of 0.82, 0.99, and 0.90, respectively. Furthermore, a biomarker panel comprising CD14, CD34, cystatin A, myocyte enhancer factor 2C (MEF2C), RGS12, and ubiquitin C (UBC) ICx could distinguish active LN from inactive LN with an AUC value of 0.85, which is comparable to or better than pathological parameters such as renal activity index (AI) and renal chronicity index (CI).

**Conclusion:**

Immunoproteomics-based discovery studies have enabled us to identify circulating immune complexes as potential biomarkers of LN.

## Introduction

Systemic lupus erythematosus (SLE) is a multifactorial and heterogeneous autoimmune disease, manifested by autoantibody production and altered type I interferon expression and regulation (1–3). About 60% of SLE patients are eventually advanced into potentially fatal lupus nephritis (LN) (4, 5). LN is a leading cause of mortality in SLE patients, and the treatment of LN has become a significant social and economic burden in the United States (6). Unfortunately, the diagnosis or disease monitoring of SLE or LN is suboptimal. The current gold standard for clinical diagnosis of LN is renal biopsy, which is invasive and may cause kidney damage (7, 8). Using serum for a liquid biopsy is minimally invasive; therefore, serum biomarkers may have great potential in the diagnosis and disease monitoring of LN patients in clinical settings. Given the heterogenetic nature and unmet needs in precision diagnosis and classification of SLE/LN patients for personalized medication, identification of novel biomarkers, particularly in the form of a biomarker panel, is of paramount importance (9). Omics studies are promising in the discovery of novel serum biomarkers which may aid in accurate diagnosis and disease monitoring of LN clinically (10, 11). Robust serum biomarkers may also be useful in developing point-of-care systems that can be used for home testing of LN.

IgG antinuclear autoantibodies (ANA) against components such as DNA and nucleoprotein are commonly found in the glomeruli and serum of individuals with LN (12). The etiology of LN involves antibody binding to multiple autoantigens (AAgs) (13). Through the Fc–FcR interaction, the intracellular or extracellular AAgs can bind to specific autoantibodies to form immune complexes (ICx), which as a double-edged sword may exert pathological effects or beneficial regulatory effects, depending on the antigen–antibody ratio, antibody subclass, and antigen subcellular location (14, 15). Therefore, disease-associated ICx, particularly AAgs, may not only contribute to the pathogenesis of the disease but also serve as disease biomarkers in autoimmune diseases such as SLE and LN.

Omics technologies, such as genomics, transcriptomics, proteomics, and metabolomics, are rapidly evolving which enable the discovery of putative biomarkers in SLE (16). In particular, single-cell RNA sequencing (scRNA Seq) allows us to investigate transcriptomic profiles at a single-cell resolution, in which the function of rare cell populations and the information on communication among different cell types can extend our understanding of the pathogenesis and heterogeneity of SLE (17). A single-cell analysis of intrinsic renal cells and infiltrating cells from patients with LN may be helpful in defining the pathways of renal injury at a cellular level (18). However, identifying a clinically useable biomarker of SLE/LN is still challenging due to two major issues: (1) heterogeneity of SLE where multifactorial pathogenesis may involve various molecular or signaling pathways in different patients; (2) difficulty in standardization of omics technological platforms and experimental systems which may result in variable preliminary data. To tackle these challenges, we aimed to combine the immunocapture-based proteomics approach with bioinformatics and existing scRNA Seq databases to pinpoint clinically useful biomarkers in SLE or LN.

## Materials and Methods

### Reagents

Protein A- or G-coated magnetic beads were purchased from Millipore Sigma (Saint Louis, MO). The native human C1Q was purchased from Abcam (Boston, MA, USA). Papain solution was purchased from Millipore Sigma, MO. The CD14, CD34, CSTA, UBC, and BST1 antibodies were purchased from R&D Systems (Minneapolis, MN, USA), the P3H1, RGS12, and GUK1 monoclonal antibodies were purchased from Santa Cruz Biotechnology (Dallas, TX, USA), and the MEF2C and PHACTR4 monoclonal antibodies were purchased from Cell Signaling Technologies (Danvers, MA, USA) and Abcam, respectively. The anti-human IgG antibody was purchased from Jackson ImmunoResearch (West Grove, PA).

### Patients and Clinical Samples

Serum samples from lupus nephritis and healthy controls were collected at the University of Texas, Southwestern Medical Center at Dallas. The samples were aliquoted and stored at -80°C. All human subject-related procedures were performed following the institutionally approved IRB protocols, and all consents were obtained before sample collection. The detailed demographics and clinical information are summarized in **Table 1**. Active lupus nephritis (LN-active) is defined as Systemic Lupus Erythematosus Disease Activity Index (SLEDAI) greater than 4 and the renal domain of SLEDAI (rSLEDAI) greater than 0. Inactive lupus nephritis (LN-inactive) is defined as SLEDAI less than 4 and rSLEDAI equal to 0. In the immunoproteomic screening study, serum samples from 3 LN patients (SLEDAI = 4, 19, and 20, respectively) or 3 healthy controls were pooled, respectively, for further experiments.

**Table 1:** Demographics and clinical characteristics of subjects.

LN patients	LN-active	LN-inactive
Total no. of subjects	49	13
Female, no. (%)	80.79%	100%
Age, mean ± SE., years	30.15 ± 8.47	37.84 ± 13.19
Ethnicity, Asian/Black/Hispanic/Hawaii/White, no.	3/23/1/1/21	1/2/0/0/10
During time from LN onset, median (interquartile), years	7 (3–10)	6 (2–12)
SLEDAI, median (interquartile)	8 (8–12)	2 (0–3)
Renal SLEDAI, median (interquartile)	4 (4–8)	4 (4–8)
No. of patients with renal SLEDAI = 0 (%)	2.04%	100%
Protein: creatinine ratio, mg/mg, median (interquartile)	2.79 (1.32–5.31)	0.12 (0.18–0.22)
Serum Cr, mg/dL, median (interquartile)	0.95 (0.71–1.85)	0.75 (0.64–0.81)
No. of patients with DNA positive (%)	33 (67.35%)	5 (38.46%)
**Treatment at time of biopsy**		
Pred (mg/d)	36 (73.47%)	6 (46.15%)
MMF (g/d)	24 (48.98%)	2 (14.29%)
HCQ (mg/d)	29 (59.18)	6 (46.15%)
**Health controls**		
Total no. of subjects	21	
Female, no. (%)	71.43%	
Age, mean ± SE., years	30.60 ± 10.04	
Ethnicity, Asian/Black/Hispanic/Hawaii/White, no	3/8/0/0/10	

### Protein A/G-Based Immunoprecipitation

40 µl of Protein A- or G-coated magnetic beads was gently mixed and washed with 0.1% PBST. 100 µl of 1:100 PBS-diluted pooled serum samples from LN or healthy controls was then mixed and incubated with the Protein A/G magnetic beads at room temperature for 30 min with gentle shaking. The immune complex bound beads were then washed three times with 50 µl PBS. A magnetic stand was used to hold the beads, while the supernatant was removed after washing. The washed beads were suspended in 50 µl of 0.1 mg/ml papain solution (0.04 M EDTA, 0.04 M L-cysteine) and incubated at 37°C for 1 h. The magnetic stand was used to remove the beads, and the resultant supernatant was transferred to a new tube, and 50 µl of 0.06 M iodoacetamide was added to terminate the reaction of papain digestion, followed by gel purification and mass-spectrometry analysis.

### C1q-Based Capture/Enrichment of Immune Complex

100 µl of 200 µg/ml native human C1q was diluted into PBS, coated onto a 96-well microplate (Thermo Fisher, Waltham, MA), and incubated overnight at room temperature. The microplate was washed three times with 150 µl PBST and blocked, and then 100 µl of 1:100 PBS-diluted pooled serum samples was added into the wells and incubated for 2 h at room temperature. The aqueous portion was removed, and the microplate was washed three times with 150 μl PBST. A 50-µl 0.1-mg/ml papain solution was added to each well and incubated at 37°C for 1 h. Then, 50 µl of 0.06 M iodoacetamide was added into the well to terminate the papain digestion, followed by gel purification and mass spectrometry analysis.

### Identification Autoantigens With Mass Spectrometry

Supernatants from the Protein A/G- or C1q-captured immune complex samples were purified by a brief running (≤10 min) of SDS-PAGE. The gel band containing samples was subjected to in-gel digestion (19). An aliquot of the tryptic digest (in 2% acetonitrile/0.1% formic acid in water) was fractionated through liquid chromatography and then analyzed by an Orbitrap Fusion™ Tribrid™ mass spectrometer (Thermo Scientific™) interfaced with a Dionex UltiMate 3000 Binary RSLCnano System. Peptides were separated onto an Acclaim™ PepMap™ C18 column at a flow rate of 300 nl/min. Gradient conditions were 3%–22% B for 40 min; 22%–35% B for 10 min; 35%–90% B for 10 min; and 90% B held for 10 min (solvent A, 0.1% formic acid in water; solvent B, 0.1% formic acid in acetonitrile). The peptides were analyzed using a data-dependent acquisition method; Orbitrap Fusion was operated with the measurement of FTMS1 at resolutions of 120,000 FWHM, scan range of 350–1,500 m/z, AGC target 2E5, and maximum injection time of 50 ms. During a maximum 3-s cycle time, the ITMS2 spectra were collected at a rapid scan rate mode, with HCD NCE 35, 1.6-m/z isolation window, AGC target 1E4, maximum injection time of 35 ms, and dynamic exclusion which was employed for 35 s. The raw data files were processed using Thermo Scientific™ Proteome Discoverer™ software version 1.4, and spectra were searched against the UniProt Homo sapiens database using the Sequest search engine v2.3.02 (Matrix Sciences, Chicago, IL, USA) run on an in-house server. Search results were trimmed to a 1% FDR for stringency and 5% for relaxed conditions using Percolator. For the trypsin digestion, up to two missed cleavages were allowed. MS tolerance was set at 10 ppm; MS/MS tolerance at 0.6 Da. Carbamidomethylation on cysteine residues was used as fixed modification; oxidation of methionine and phosphorylation of serine, threonine, and tyrosine were set as variable modifications. The sum of scores of individual peptides for identified proteins was used to identify proteins from the immune complex.

### Annotation Enrichment Analysis

A total of 77 candidate proteins with protein accession numbers were converted into gene names with DAVID (https://david.ncifcrf.gov) and UniProt (https://www.uniprot.org) within Homo sapiens species. The functional enrichment gene-set analysis for three GO (Gene Ontology) sub-ontologies was performed with ClusteProfiler 4.0 (20) with a p-value cutoff of 0.05 and the “Benjamini–Hochberg” p-Adjust-value method. The “Disease class,” “Up tissue,” and “Kyoto Encyclopedia of Genes and Genomes (KEGG) Pathway” annotations were retrieved from DAVID (21).

### Gene Expression Analysis of the Autoantigens in 6 Genomic-Level Databases

Six publicly available SLE or LN databases (**Supplementary Table 3**) were downloaded to cross-validate the autoantigens discovered using immunoproteomics in the present study. In the 6 databases, 3 major genomic technologies were used, including single-cell RNA Sequencing (scRNA Seq), total RNA sequencing (RNA-Seq), and gene expression microarray (RNA-Array), to eliminate bias generated by single technology or sample type. The differentially expressed genes (DEGs) were defined as adjusted p-values (p-adjust) smaller than 0.05 between SLE/LN and healthy controls. In the two scRNA databases, Seurat V4 was used to generate an expression matrix, PCA dimension reduction, and cell cluster as described previously (22). Each cluster cell type was identified with reference-based package SingleR (23) and verified with canonical markers. Then, the DEGs were discovered by DEsingle (24). For two RNA databases, processed count files were directly obtained from GEO, and the DEGs were discovered by DESeq2 (25). The databases from the two RNA-Array were downloaded and standardized, and the DEGs were screened using the GEOquery and Limma (26, 27).

### Validation of Immune Complex in LN Using ELISA

ICx in the serum of LN and controls was measured using an in-house-developed ELISA kit. Briefly, to capture antigen-specific ICx, commercial monoclonal antibodies (tested by Western blot) against each autoantigen were coated onto the Immulon 2 HB flat-bottom microtiter plates (Thermo Fisher, MA) overnight at 4°C and then blocked. Serum samples were diluted into 1:100 in reagent diluent, added into the wells, and incubated for 2 h at room temperature. Anti-human IgG antibody (Jackson ImmunoResearch, PA) was diluted into 1:20,000 and added into the wells and incubated for 1.5 h at room temperature followed by color development. The ELISA signal was read by an Epoch plate reader (BioTek, Winooski, VT) at 450 nm, and the blank was subtracted from the OD450 readings of the samples.

### Statistical Analysis

All data were analyzed, plotted, or visualized with R 4.1.0 language or ggplot2 (28) package unless stated otherwise. Biomarker-level group-wise comparisons of statistical significance (p-values) were analyzed using the Wilcoxon test. Principal component analysis (PCA) was conducted to transform the serum levels of ICx into uncorrelated principal components, and only the first two components were used for the dot plot. Sensitivity (true positive ratio), 1-specificity (false positive ratio), and area under the curve (AUC) values were determined by receiver-operating characteristic (ROC) analysis using a pROC package (29). The least absolute shrinkage and selection operator (LASSO) (30) was used to evaluate and select candidate serum ICx as the best panel of biomarkers to distinguish LN from HC or LN-active from LN-inactive with the largest value of lambda under 3-fold cross-validation. The correlation between serum ICx levels and clinical parameters was determined by Spearman’s correlation coefficient.

## Results

### Immunoproteomics-Based Discovery of Novel ICx in the Serum of LN

**Figure 1:**
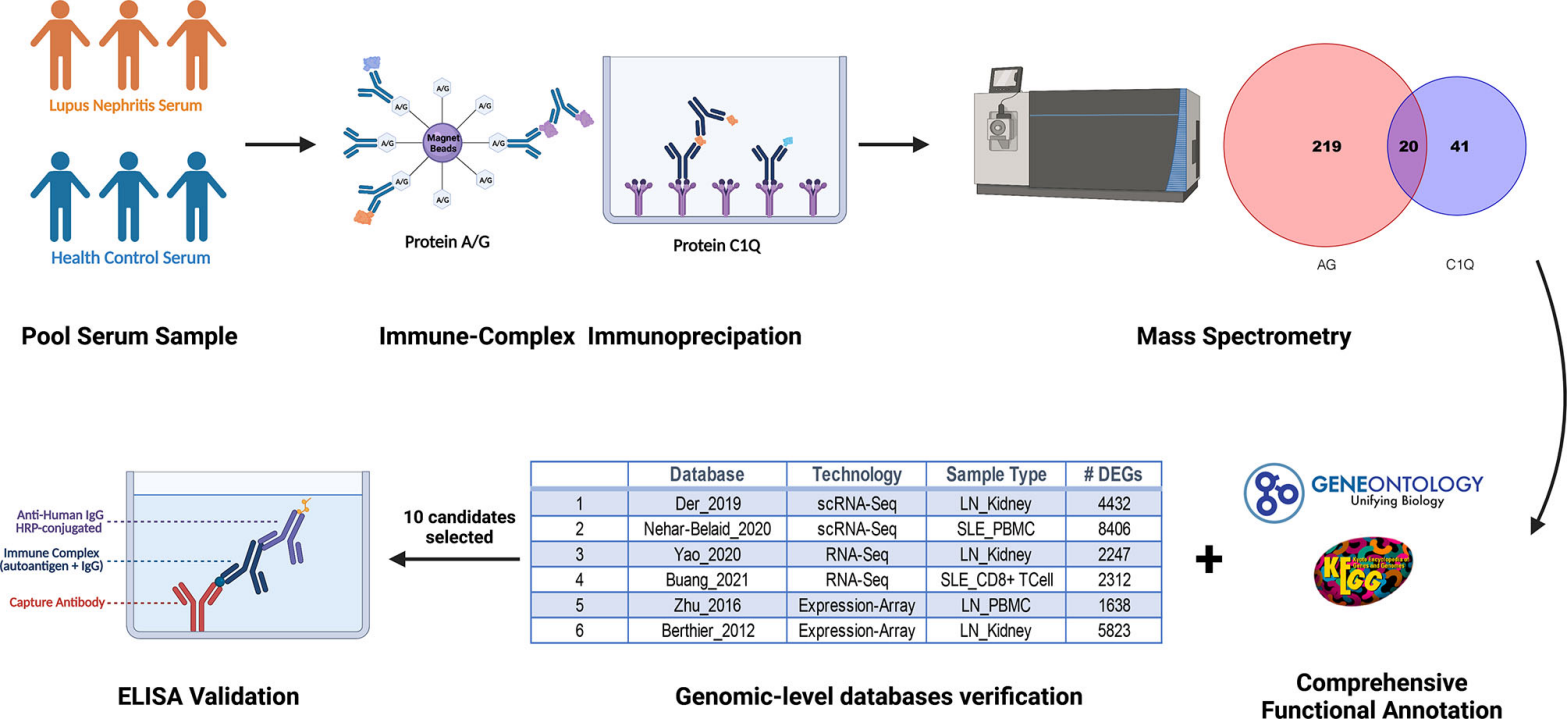
Schematic diagram of discovery and validation of immune complex (ICx) in lupus nephritis (LN) using the immunoproteomic approach. Serum samples from patients with LN or healthy control (n =3 per group) were collected and pooled for Protein A/G or C1q-based ICx capture, followed by the identification of the proteins using mass spectrometry. Candidate ICx were further validated in a larger cohort of LN patients and healthy controls using ELISA. This figure was created using a graphing program from BioRender.com.

**Figure 2:**
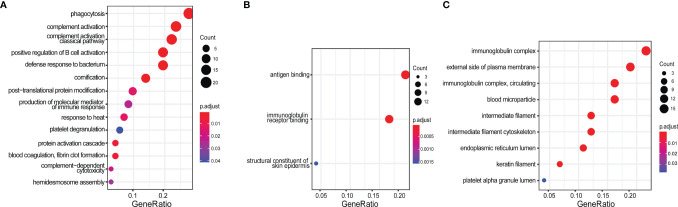
Gene Ontology (GO) enrichment analysis of 77 protein candidates discovered by immunoproteomics. **(A)** Biological process, **(B)** molecular function, and **(C)** cellular component. The gene ratio was calculated by k/n, where k = the size of the unique genes annotated in the specific gene set (e.g., immune complex); n = size of the input genes of interest (e.g., all 77 protein-encoding genes identified from this study).

To discover novel ICx that are differentially expressed in SLE or LN, we designed two strategies to capture ICx, as illustrated in **Figure 1**, showing Protein A/G magnetic beads or C1q-based capture of immunoglobulins or the antigen–antibody complex. Pooled serum samples from 3 LN patients or 3 health controls were used to capture and enrich ICx. As shown in **Supplementary Figure 1**, SDS-PAGE was used to visualize the immunocaptured products. After gel purification and in-gel digestion, the peptides were analyzed using liquid chromatography and tandem mass spectrometry (LC-MS/MS). In total, 239 and 61 immunoglobulin or immunoglobulin-binding proteins were discovered *via* Protein A/G and C1q, respectively. Based on the cumulative protein score of each protein, we ended up with identifying 52 (Protein A/G method) and 27 (C1Q method) proteins which were only detectable in the serum of LN but not healthy controls by mass spectrometry, as shown in **Supplementary Table 1** and **Supplementary Table 2**. Among the 77 unique proteins identified, TTR and KRT14 were found in both Protein A/G and C1q procedures. Gene Ontology (GO) enrichment analyses were performed to investigate the enrichment function of these proteins that are expressed in the LN patients, as shown in **Figure 2**. In the biological process (**Figure 2A**), the protein functions of complement activation and B cell activation were significantly enriched which indicated that the 77 proteins may be involved in forming an immune complex during disease development and contribute to the pathogenesis of SLE or LN. The most significant cellular component (CC), immunoglobulin complex, represents 16 immunoglobulins which consist of 11 IgH, 3 IgK, and 2 IgL. Several IgH were found associated with B cell receptor (BCR) analysis of immune-mediated diseases, such as IGHV4-34 and IGHV4-59, which are highly expressed in SLE (31).

### Functional Relevance of Immuno-Captured Proteins to SLE or LN

Given that the 77 proteins captured through immunoprecipitation were only detectable in LN but not healthy controls using the immunoproteomic approach, we assumed they may be functionally relevant to SLE or LN. To confirm this, we performed a bioinformatics analysis to determine which proteins may be involved in the disease course of SLE or LN and may potentially serve as a disease biomarker. As shown in **Supplementary Table 1** and **Supplementary Table 2**, among the 77 proteins, 32 of them are highly abundant proteins including keratins, histones, complement system proteins, and albumin and immunoglobulin family proteins which have already been discussed extensively in previous studies (32–36). Therefore, they were eliminated from further analysis in the current study. For the remaining 45 proteins, we performed the following bioinformatics analysis: Database for Annotation, Visualization and Integrated Discovery (DAVID), Kyoto Encyclopedia of Genes and Genomes (KEGG), and Gene Ontology Biological Processes (GO-BP), to determine the relevance of the candidate autoantigens (AAgs) to SLE or LN.

**Figure 3:**
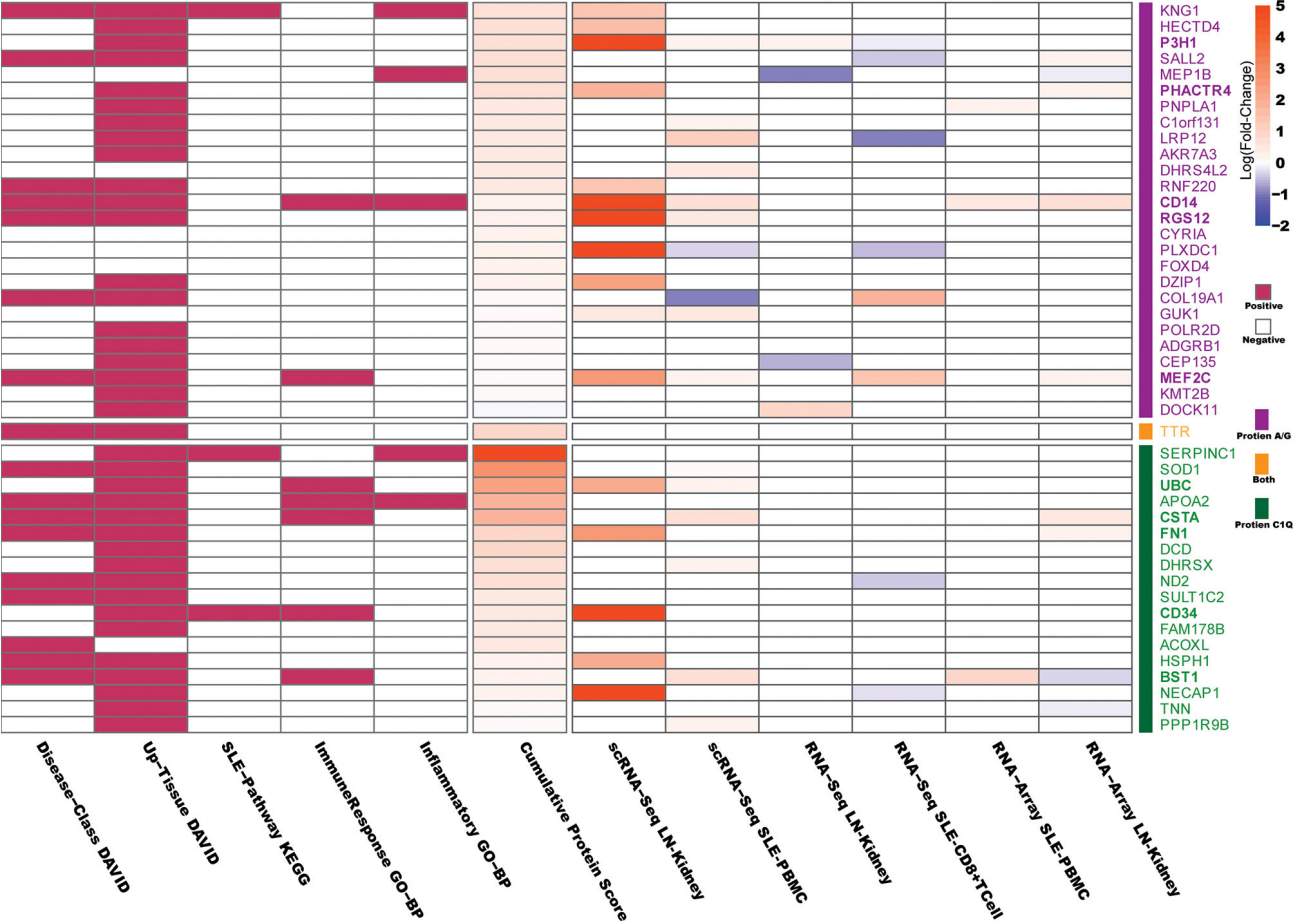
Bioinformatics analysis of the protein candidates. The Database for Annotation, Visualization and Integrated Discovery (DAVID), Kyoto Encyclopedia of Genes and Genomes (KEGG), and Gene Ontology Biological Processes (GO-BP) were used to determine the relevance of the differentially expressed protein candidates to SLE or LN. In DAVID analysis, “renal” or “immune” disease-related proteins or the proteins expressed in SLE-affected tissues (such as skin, muscle, bone, joints, kidney, spleen and brain) were considered “positive.” In KEGG analysis, proteins involved in the “Systemic lupus erythematosus - Homo sapiens (human)” KEGG pathway and its eight related pathways were considered “positive.” In GO-BP analysis, “immune response” and “Inflammatory” annotated proteins were considered “positive.” The “Cumulative Protein Score” is the sum of the peptide scores for each protein identified in LN, which were then normalized to be in the range of 0~5. The Differentially Expressed Genes (DEGs) corresponding to the 45 candidate autoantigens that appeared in six published SLE/LN databases are indicated in the right-hand column of the heatmap, where the filled color represents the log (fold-change) values (p.adjust < 0.05) as indicated in the scale bar. The details of the six databases are presented in **Supplementary Table 3**.

In DAVID analysis, we collected 5 features of DAVID functional annotation as described previously (21). As SLE is aberrant in immune function and causes end-organ damage, particularly renal damage (37), the “renal” or “immune” disease-related proteins or the proteins expressed in SLE-affected tissues (such as skin, muscle, bone, joints, kidney, spleen, and brain) were considered “positive” and are indicated in red in **Figure 3**. In KEGG analysis, the proteins involved in “Systemic lupus erythematosus - Homo sapiens (human)” KEGG pathway (https://www.genome.jp/entry/pathway+hsa05322) and its eight related pathways (hsa04060, hsa04514, hsa04610, hsa04612, hsa04630, hsa04660, hsa04662, and hsa04670) were considered “positive” and are indicated in red in **Figure 3**. In GO-BP analysis, the “immune response” and “Inflammatory” annotated proteins were considered “positive” and are indicated in red in **Figure 3**. The “Cumulative Protein Score” is the sum of the peptide scores for proteins in LN patients, which was normalized to be in the range of 0~5.

Next, we used 6 published gene expression databases of SLE or LN as detailed in **Supplementary Table 3**, to determine if the genes that encode the autoantigens were differentially expressed in SLE/LN compared to healthy controls. The 6 databases were derived based on data from single-cell RNA sequencing, total RNA sequencing, or gene expression microarray on human SLE/LN genomes from various cell or tissue types (38–43). Differentially expressed genes (DEGs) were defined as adjusted p-values (p-adjust) less than 0.05 between SLE/LN and healthy controls. As shown in **Figure 3**, the filled color represents the log (fold-change) values (p.adjust < 0.05) as indicated in the scale bar.

Two general criteria were used to determine which candidate proteins (antigens) would be selected to move forward for the next phase of validation studies: 1) the protein received at least one “positive” in the comprehensive functional annotations (DAVID/KEGG/GO, **Figure 3**) and 2) the protein-coding genes are “upregulated” in two genomic databases (**Supplementary Table 3**) or highly upregulated in one genomic database. As a result, 10 candidate proteins satisfied both criteria 1 and criteria 2 and were selected for validation.

### Validation of Selected Autoantigens/Immune Complexes

A total of 10 candidate AAgs including BST1, CD14, CD34, CSTA, FN1, MEF2C, P3H1, PHACTR4, RGS12, and UBC were selected for validation. Given the fact that the immune-capture process was designed to capture either immunoglobulins or the antigen–antibody complex with Protein A/G- or C1Q-based methods, the proteins/antigens identified by mass spectrometry were presumably the “bound-form” and originated from the antigen–antibody complex. Therefore, validating serum ICx or bound-form antigens is more reasonable than validating the free-form antigens. We then used monoclonal antibodies against each specific AAg to coat the ELISA plate, and the corresponding ICx were captured and detected on the plate using a secondary antibody, anti-human IgG conjugated with horseradish peroxidase (HRP). In the validation study, we used an independent cohort of 62 LN patients and 21 healthy controls. Detailed patient demographics and clinical characteristics information are presented in **Table 1**. The LN patients were divided into LN-active and LN-inactive based on SLEDAI and rSLEDAI index, as detailed in *Methods*.

**Figure 4:**
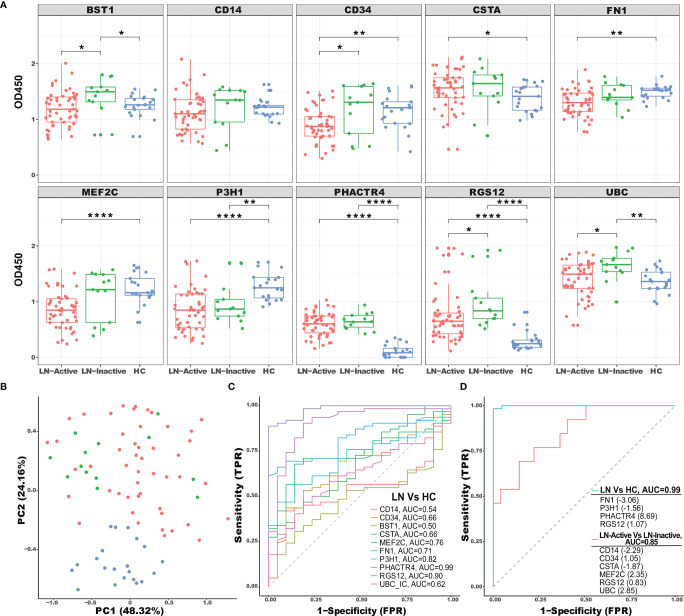
ELISA validation of selected ICx in an independent cohort of LN patients. **(A)** A total of 83 serum samples from healthy controls (N = 21, blue), inactive LN (N = 13, green), and active LN (N = 49, red) were tested by sandwich ELISA for immune complex levels of BST1, CD14, CD34, CSTA, FN1, MEF2C, P3H1, PHACTR4, RGS12, and UBC. Asterisks designate the level of statistical significance: *p < 0.05, **p < 0.01, ****p < 0.0001, using the Wilcoxon test. **(B)** Principal component analysis (PCA) of 10 ICx among HC, inactive LN, and active LN individuals. The two first principal components (PC1, PC2) were plotted. **(C)** The discriminatory abilities of the 10 ICx in distinguishing LN patients from healthy control were examined using ROC analysis. **(D)** Using LASSO regression analysis, the ICx were combined into panels to distinguish LN patients from healthy control or active LN from inactive LN.

As shown in **Figure 4A**, the BST1, PHACTR4, RGS12, and UBC-specific ICx were significantly upregulated in LN-inactive patients, compared to the healthy controls. Furthermore, BST1, CD34, RGS12, and UBC-specific ICx were significantly elevated in LN-inactive compared to LN-active patients (**Figure 4A**). Surprisingly, P3H1 immune complex levels were significantly downregulated in LN-inactive patients, compared to the healthy controls. Also, FN1- and MEF2C-specific ICx were decreased in LN-active compared to healthy controls. This inconsistency with the screening data may be due to the heterogeneity of SLE and the relatively smaller sample size in the screening cohort. Also, some of the commercial monoclonal antibodies may not be optimal (e.g., affinity or epitopes) in maximally capturing serum ICx by ELISA. In addition, it is conceivable that serum CD34 and MEF2C ICx levels may respond to drug treatment, because they were significantly lower in the LN-active group compared to the LN-inactive group following drug treatment (**Supplementary Figure 2**). However, no significant responses to drug treatment were observed in the other ICx as well as in anti-dsDNA autoantibodies (**Supplementary Figure 2**).

To uncover the distribution patterns of the 10 ICx in an unbiased manner, dimension reduction principal component analysis (PCA) was performed using the ELISA validation data. The percentage of variance for the first and second principal components (PCs) were 48.32% and 23.16%, respectively, which were used to map both LN and HC samples (**Figure 4B**). The HC samples were clearly separated from LN patients implying the distinction of their ICx expression patterns. The discriminative capabilities of 10 ICx for LN vs. HC were evaluated with ROC analysis (**Figure 4C**). P3H1, PHACTR4, and RGS12 ICx outperform other markers with AUC values greater than 0.8. Furthermore, when individual ICx were combined into a biomarker panel with the LASSO model, the discriminative ability was significantly improved (**Figure 4D**). In the LN vs. HC group, the AUC value was 0.99, where the greatest positive and negative contributing variables were PHACTR4 and FN1, respectively. In the LN-active vs. LN-inactive group, 4 positive (CD34, MEF2C, RGS12, and UBC) ICx and 2 negative (CD14 and CSTA) ICx contributing variables were identified using the LASSO model. When combined into a panel, these biomarkers can discriminate LN-active from LN-inactive with an AUC value of 0.85. In comparison, when using clinical parameters to discriminate LN-active from LN-inactive, the AUC values were 0.81 for renal chronicity index (CI), 0.62 for renal activity index (AI), 0.58 for white blood cell counts (WBC), and 0.91 for proteinuria. Hence, the LASSO-derived serum biomarker panel (CD14, CD34, CSTA, MEF2C, RGS12, and UBC ICx) outperformed the renal pathology indices such as AI and CI.

### Serum ICx May Reflect Pathological Disease Activity

**Figure 5:**
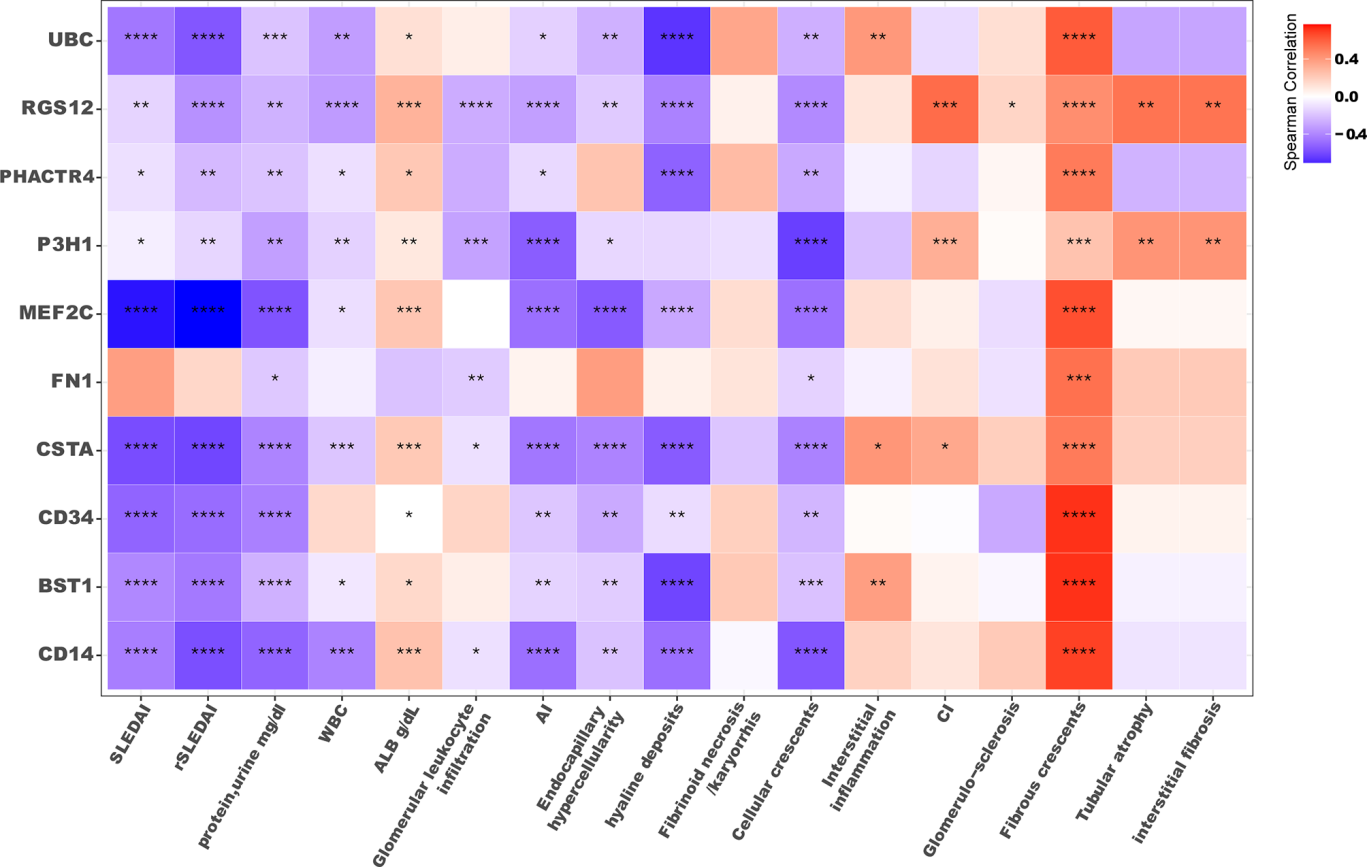
Correlation plot of serum ICx levels with clinical parameters. The color filled in each square represents Spearman’s correlation coefficient value, and the corresponding significance level was indicated with asterisks: *p < 0.05, **p < 0.01, ***p < 0.001, ****p < 0.0001. SLEDAI, SLE disease activity index; rSLEDAI, renal SLE disease activity index; WBC, white blood cell count; ALB, the amount of albumin in blood; AI, renal pathology activity index, CI, renal pathology chronicity index.

Next, we determined if serum ICx were associated with clinical and pathological parameters and if they had a diagnostic value in reflecting renal pathological change without the need of renal biopsy. As shown in the correlation heatmap in **Figure 5**, most serum ICx exhibited a significant negative correlation with SLEDAI, rSLEDAI, WBC, and proteinuria. Interestingly, serum ICx levels exhibited a negative correlation with AI but a positive correlation with CI, as well as the individual components of AI and CI. This is consistent with the fact that serum ICx was lower in LN-active compared to LN-inactive patients. Besides, the longitudinal studies indicated that BST1, CSTA, and UBC serum ICx levels changed over two time points, which could track with changes in SLEDAI and/or rSLEDAI (**Supplementary Figure 3**).

### Gene Expression of Autoantigens in Various Cell Types in SLE

**Figure 6:**
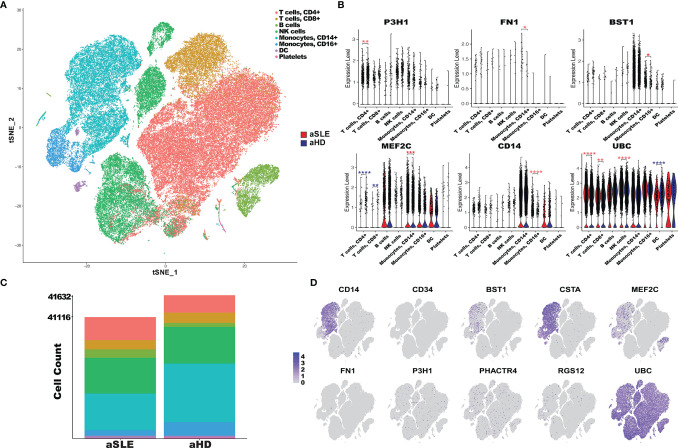
PBMC scRNA expression of autoantigen candidates in SLE patients and healthy controls. **(A)** tSNE plot of 82,748 cells across 23,257 gens from the PBMC sample of 8 adult SLE patients (aSLE) and 5 adult healthy controls (aHD). Clusters were derived from PCA reduction with the first 18 dimensions, and the clusters’ cell type was identified with SingleR reference databases and canonical markers. **(B)** The bar plot represents the cell abundance of 8 clusters across aSLE (41,116 cells) and aHD (41,632 cells) groups. **(C)** Violin plots of PBMC scRNA expression in 8 cell type clusters in aSLE (red) and aHD (cyan). The autoantigens’ differential expression levels in each cluster were determined using the Wilcoxon test. The asterisks designate the level of significance: *p < 0.05, **p < 0.01, ***p < 0.001, ****p < 0. 0001, and increased or decreased expression in aSLE was colored in red and cyan, respectively. **(D)** Feature plots of 10 autoantigens; each dot represents one cell, and the color intensity indicates the gene expression level of autoantigen in the cells, from gray to purple.

To determine if these AAg-encoding genes are differentially expressed in various cell types in LN, we examined the gene expression of the AAgs at a single-cell resolution using a database of scRNA sequencing of PBMC from 8 SLE adult patients and 5 healthy controls (42). As shown in **Figure 6A**, 8 different cell types were identified from 82,748 cells and each cell population in the SLE and HC groups is displayed in **Figures 6A, B**. CD14, BST1, CSTA, and MEF2C were found to have an enriched gene expression in CD14+ or CD16+ monocytes, and MEF2C was also highly expressed in SLE B cells compared to healthy controls. Furthermore, the significance test using Wilcoxon comparison between SLE and HC in different cell types is presented in **Figure 6C**. P3H1, PHACTR4, and UBC were ubiquitously expressed in all cell types (**Figure 6D**). In the CD4+ T cell cluster, P3H1 and UBC were found upregulated, whereas MEF2C was downregulated in the SLE group, compared to healthy controls. In monocytes, CD14 were highly expressed in CD16+ cells, and MEF2C and FN1 were upregulated in CD14+ cells in SLE compared to healthy controls. UBC exhibited overall the strongest expression across all cell types, and it was significantly upregulated in CD4+ T cells, CD8+ T cells, and natural killer (NK) cells in SLE compared to HC. However, it was significantly downregulated in dendritic cells (DCs) in SLE, compared to HC. The gene expression profiles of these AAgs were also examined in 1,496 cells from LN renal tissues using the scRNA Seq database (details are shown in **Supplementary Figure 4**). The results demonstrate that MEF2C were highly expressed in leukocytes, endothelial cells, and mesangial cells; FN1 exhibited an overall higher gene expression in renal cells compared to PBMCs; UBC was also found highly expressed across all cell types in the kidney, which is consistent with the PBMC data.

In summary, we found that PHACTR4, RGS12, UBC, CSTA, and BST1 are concordantly elevated in both our ICx assays and the public gene expression database, as detailed in **Figure 4** and **Supplementary Table 3**.

## Discussion

Experimental disease models of glomerulonephritis (GN) and vasculitis have verified the potential of circulating ICx (antigen–antibody complexes) in causing disease (44). ICx are responsible for the GN of lupus and also contribute to the pathogenesis of other manifestations in SLE (45, 46). ICx play a complicated role in LN, either by depositing on vessels/tissues to cause pathological effects or by interacting with receptors on immune cells to initiate immunological regulations (15). Several assays that indirectly measure circulating ICx have been developed to evaluate ICx-mediated inflammation in patients with SLE; however, currently available assays are insufficient to reliably and reproducibly detect ICx (47). In this study, we employed both Protein A/G magnetic beads and microplate-based C1q to capture, enrich, and purify ICx from lupus serum. In this particular study, it seems that the Protein A/G-based approach is more efficient (239 proteins) in capturing ICx in SLE, compared to the C1q method (61 proteins). This may be due to the affinity differences between Protein A/G and C1q in binding ICx.

The DEGs from six transcriptomics SLE/LN databases were informative to understand the functional aspects of the autoantigens and to select the most relevant proteins/ICx for further validation. However, given the fact that the 6 databases were developed using different technologies, this inevitably poses a challenge to integrate all data which requires statistical framework and tremendous computational resources. To address this challenge, the DEGs were separately calculated using mainstream methods for each technology (24–26) and then aggregated and visualized with a heatmap. The discrepancy in gene expression profiles of the same genes across six databases may be due to the heterogeneity of LN or platform differences. For example, LRP12 and COL19A1 gene expression changes were found to be opposite in SLE between a scRNA database and an RNA database. In another scenario, PLXDC1 expression levels were different in SLE between PBMCs and kidney cells at a single-cell resolution, which suggested that the context of cells is critical.

It is worth noting that PHACTR4 ICx were significantly elevated in LN patients compared to HC (AUC = 0.99), and consistent transcription profiles of this molecule were also found across two kidney databases, based on scRNA Seq and gene expression microarray. Interestingly, previous studies have identified PHACTR4 as a tumor suppressor in several cancers, with functions in PP1 localization and Rb dephosphorylation (48). The aberrant gene expression in the kidney and formation of ICx may contribute to LN pathogenesis. The regulator of G-protein signaling 12 (RGS12) ICx was found upregulated in LN, compared to HC. Interestingly, higher levels of RGS12 ICx were detected in the LN-inactive compared to LN-active group. Furthermore, serum RGS12 ICx exhibited a positive correlation with chronicity index (CI), as well as glomerulosclerosis, fibrous crescents, tubular atrophy, and interstitial fibrosis. In a study of rheumatoid arthritis, a significant association was found between RGS12 genetic variation with the response to an immunosuppressive drug (49). These data suggest that RGS12 may be involved in the pathogenesis of SLE and other rheumatic diseases. Notably, P3H1 ICx was the only one found significantly downregulated in LN (AUC = 0.82) compared to HC. P3H1 mRNA was found upregulated in 3 gene expression databases and downregulated in one database. In the PBMC scRNA Seq database, transcriptomic P3H1 was significantly upregulated in CD4+T cells. P3H1 is responsible for the 3-hydroxyproline posttranslational modification of type I collagen; if defective, it may cause renal pathology (50). Therefore, P3H1 may be involved in renal pathology and contribute to the pathogenesis of LN. In addition, the fact that serum CD34 and MEF2C ICx but not anti-dsDNA autoantibody levels responded to drug treatment (**Supplementary Figure 2**) may indicate that novel serum ICx may be a better biomarker in assessing drug responses in LN. It is important to highlight that PHACTR4, RGS12, UBC, CSTA, and BST1 are concordantly elevated in both our ICx assays and the public gene expression database, as detailed in **Figure 4** and **Supplementary Table 3**. The reason that the two ICx enrichment methods resulted in different protein profiles may be due to structurally different binding sites in the antibodies and different binding kinetics/affinities for ICx. As indicated in the literature, C1q binds to CH2 domains of the Fc (51), whereas protein A and protein G mainly bind to the CH2-Ch3 domains (52, 53). Thus, it is likely that protein A/G and C1q may select and capture different antibodies in the ICx, which were characterized by mass spectrometry-based sequencing of the protein/antigen.

The caveats of this study include (1) the relatively small sample size especially in the LN-inactive group and (2) the lack of various disease controls such as other autoimmune diseases and other kidney diseases due to the limited access to these samples. In future studies, the increase of the sample size in the LN-inactive group may aid to confirm if and why serum ICx were lower in LN-active compared to LN-inactive. The inclusion of disease controls may be helpful in better establishing the specificity of these ICx biomarkers in LN.

## Conclusion

Immunoproteomics-based discovery studies have entabled us to identify promising immune complexes in LN, which are associated with clinical parameters including renal pathology indices. These ICx may be useful in diagnostics, disease monitoring, and/or assessing drug responses in LN contingent upon further validation.

## Data Availability Statement

The mass spectrometry proteomics data have been deposited to the ProteomeXchange Consortium *via* the PRIDE (1) partner repository with the dataset identifier PXD031069.

## Ethics Statement

The studies involving human participants were reviewed and approved by University of Houston IRB. The patients/participants provided their written informed consent to participate in this study.

## Author Contributions

TW conceived this study. CT, MF, GT, and TZ performed the experiments and/or collected the data. CT, MF, GT, SZ, BY, YL, TZ, RS, CM, and TW were involved in the data analysis or data interpretation. CT and TW wrote the manuscript. SZ, BY, and CM edited the manuscript. All authors contributed to the article and approved the submitted version.

## Funding

This work was supported by an NIH grant R01AG062987 to TW. This work was partly supported by George M. O’Brien Kidney Research Core Center (National Institutes of Health P30DK079328) grant to RS.

## Conflict of Interest

The authors declare that the research was conducted in the absence of any commercial or financial relationships that could be construed as a potential conflict of interest.

## Publisher’s Note

All claims expressed in this article are solely those of the authors and do not necessarily represent those of their affiliated organizations, or those of the publisher, the editors and the reviewers. Any product that may be evaluated in this article, or claim that may be made by its manufacturer, is not guaranteed or endorsed by the publisher.

## Abbreviations

ICx, immune complexes; LN, lupus nephritis; HC, healthy control; GN, glomerulonephritis; SLE, systemic lupus erythematosus; ROC, receiver operating curve; AUC, area under the curve; AI, renal Activity Index; CI, renal Chronicity Index; GO, Gene Ontology; BCRs, B cell receptors; scRNA Seq, single-cell RNA sequencing; DEGs, differentially expressed genes; PCA, principal component analysis; KEGG, Kyoto Encyclopedia of Genes and Genomes; DAVID, Database for Annotation, Visualization and Integrated Discovery; LC-MS/MS, liquid chromatography and tandem mass spectrometry.
