# Supplementary material for: Discovery of Novel Circulating Immune Complexes in Lupus Nephritis Using Immunoproteomics
Source: Front Immunol. 2022 Mar 24;13:850015. doi: 10.3389/fimmu.2022.850015 (PMC8996714; doi:10.3389/fimmu.2022.850015)
Supplement: Supplementary file 1 [file DataSheet_1.pdf]

Supplementary Table 1. The 52 serum autoantigens detected only in LN patients through Protein A/G based immune complex capture and mass spectrometry

Accession	Gene	MW [kDa]	PSMs	Cumulative protein score	calc. pI
Q6N089	IGHV3-15	51.69	134	364.61	7.93
Q15323	KRT31	47.21	70	144.41	4.88
P78385	KRT83	54.16	71	121.71	5.64
O76009	KRT33A	45.91	52	104.15	4.82
C4AMA3	KRT34	44.69	42	82.42	4.78
O76013	KRT36	52.21	36	71.34	4.94
P78386	KRT85	55.77	46	62.31	6.55
Q701L7	KRT82	56.61	23	46.60	6.74
Q0ZCI9	IGHV3-33	14.13	9	22.66	7.94
P02533	KRT14	51.53	13	22.14	5.16
A2NB45	IGKV2-28	12.38	12	20.53	8.48
Q9UL84	IGHV3-30	13.57	7	18.11	8.46
Q65ZC9	SCFV	25.55	8	15.94	9.11
P04433	IGKV3D-11	12.57	11	15.51	4.96
A2J1M2	IGHV1-69	10.54	8	14.64	9.17
A8K5T0	CFH	138.86	7	12.17	6.71
A8MTN3	KRTAP2-2	12.95	3	7.43	7.81
A2KLM6	IGHV5-51	10.54	3	7.43	7.24
A6XMH1	TTR	14.95	2	4.21	5.76
A2NXP8	IGHV3-64	13.82	2	4.12	9.72
C9JEX1	KNG1	43.79	2	4.09	6.43
Q9Y4D8	HECTD4	439.07	2	4.08	6.19
E2QRI1	P3H1	23.07	1	3.76	5.44
B4DED3	SALL2	88.91	1	3.71	6.51
Q16820	MEP1B	79.52	1	3.69	5.74
Q5NV88	IGLV6-57	10.64	3	3.58	4.78
Q8IZ21	PHACTR4	78.16	1	3.52	6.62
Q8N8W4	PNPLA1	57.84	1	2.94	8.07
B4E0F7	C1orf131	26.66	1	2.86	9.33
E5RH57	LRP12	15.51	1	2.83	6.28
O95154	AKR7A3	37.18	1	2.80	7.15
D5KJA2	DHRS4L2	15.17	1	2.74	7.96
Q5VTB9	RNF220	62.73	1	2.73	6.04
D6RFL4	CD14	23.30	1	2.64	5.26
E7EMN9	RGS12	61.05	1	2.48	9.11
B3KRB1	CYRIA	20.47	1	2.23	7.78

A2NYU8	IGHV4-59	12.67	1	2.19	9.20
C9JWQ3	PLXDC1	25.30	1	2.10	6.60
P01775	IGHV3-23	12.85	1	2.03	8.48
Q3MI95	FOXD4	43.07	1	2.02	9.42
C9JDC7	DZIP1	30.47	1	2.01	8.46
Q14993	COL19A1	115.15	1	1.90	8.32
Q96IN2	GUK1	20.32	1	1.88	6.21
Q5NV79	IGLV4-60	10.67	1	1.86	5.81
Q587J0	POLR2D	6.63	1	1.82	4.83
A8K9J7	H2BC5	13.99	2	1.70	10.32
A2NB43	IGHV4-34	13.04	2	1.70	9.41
E5RG74	ADGRB1	126.61	2	1.64	7.90
Q66GS9	CEP135	133.41	1	1.64	6.21
D7F7N5	MEF2C	44.91	1	1.63	9.22
A8MVY7	KMT2B	288.16	1	1.61	8.24
Q5JSL3	DOCK11	237.52	1	1.61	7.74

Supplementary Table 2. The 27 serum autoantigens detected only in LN patients through C1Q based immune complex capture and mass spectrometry.

Accession	Gene	MW [kDa]	PSMs	Cumulative protein score	calc. pI
A8K9P0	ALB	69.22	60	99.50	6.28
Q96KK5	H2AC12	13.90	23	76.76	10.89
B4E1T1	KRT5	58.81	19	32.70	5.97
P01857	IGHG1	36.08	15	27.80	8.19
P02533	KRT14	51.53	14	23.72	5.16
P01834	IGKC	11.60	9	23.55	5.87
Q8TCE1	SERPINC1	29.07	8	17.39	8.81
A8MST3	SOD1	13.90	3	10.25	6.11
Q96C32	UBC	34.28	4	8.89	7.53
E7EU73	C3	122.51	3	8.44	6.76
P02652	APOA2	11.17	4	7.75	6.62
P01859	IGHG2	35.88	5	7.53	7.59
P01040	CSTA	11.00	2	7.21	5.50
B7ZLF0	FN1	239.47	3	4.53	5.88
P81605	DCD	11.28	2	4.40	6.54
C9JRH1	DHRSX	13.68	1	4.06	9.28
Q14X33	ND2	14.77	1	3.80	9.88
C9JSR0	SULT1C2	14.62	2	3.41	6.29
B4DG27	CD34	27.04	1	3.31	7.64
E9PFC2	FAM178B	72.80	1	2.85	5.43
Q9NUZ1	ACOXL	61.76	2	2.74	8.53
A6XMH1	TTR	14.95	1	2.63	5.76
Q5TBM3	HSPH1	13.17	1	2.43	5.07
Q1XII0	BST1	29.31	1	2.31	6.77
B4E160	NECAP1	15.13	1	2.21	6.73
B7ZM70	TNN	124.32	1	1.89	5.78
B3KNT0	PPP1R9B	35.11	1	1.67	4.88

MW [kDa], molecular weight of the target protein; PSMs, the total number of identified peptide sequences (peptide spectrum matches) for the protein, including those redundantly identified; Cumulative protein score, the sum of the scores of the individual peptides for identified protein in LN patient. calc. pI, the theoretically calculated isoelectric point.

Supplementary Table 3. Six SLE/LN genomic databases used in this study.

	Database	Technology	Platform	# Patient	# Health	# DEGs	Accession Number	Database Name
1	Der_2019[1]	scRNA-Seq	Fluidigm C1 HT + Illumina NextSeq500	21	3	4432	SDY997	ImmPort
2	Nehar-Belaid_2020[2]	scRNA-Seq	10X Genomics + Illumina HiSeq 4000	8	5	8406	phs002048.v1.p1	dbGaP
3	Yao_2020[3]	RNA-Seq	Illumina HiSeq 2500	3	3	2247	GSE157293	GEO
4	Buang_2021[4]	RNA-Seq	Illumina HiSeq 2500	34	14	2312	GSE97264	GEO
5	Zhu_2016[5]	Expression-Array	Illumina HumanHT-12 V4.0 expression beadchip	15	25	1638	GSE81622	GEO
6	Berthier_2012[6]	Expression-Array	Affy_HGU133A_CDF_ENTREZG_10	32	15	5823	GSE32591	GEO

Note: Sample type used in each study: 1. Kidney, 2. PBMC, 3. Kidney, 4. CD8+ T cells, 5. PBMC and 6. Kidney.

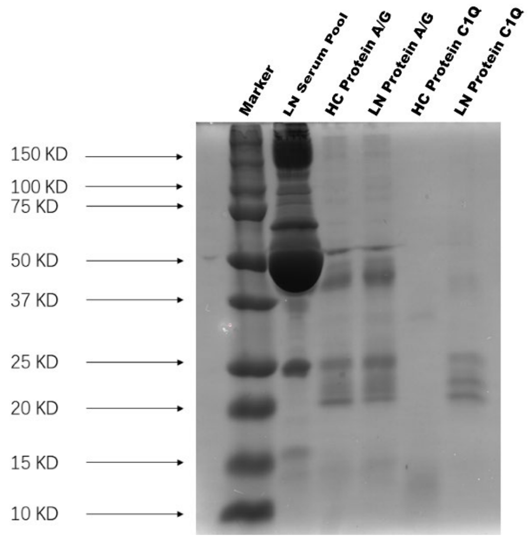

Supplementary Figure 1. SDS-PAGE analysis of the immuno-captured proteins/immune complex from serum samples of lupus patients or healthy controls using Protein A/G or C1q immunoprecipitation. Pooled serum samples from lupus patients (N=3) or healthy controls (N=3) were used in each immunoprecipitation experiment.

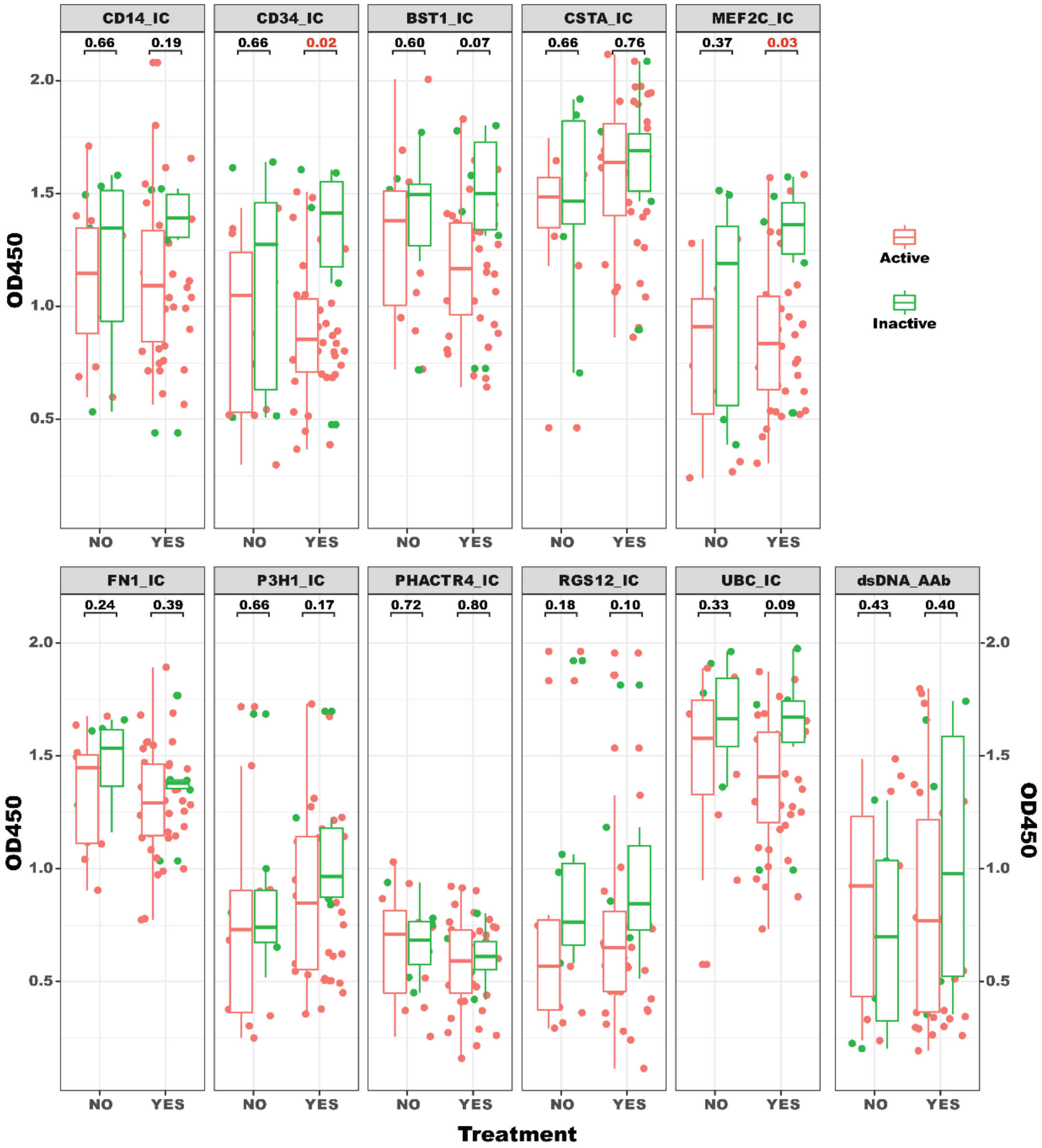

Supplementary Figure 2. Drug treatment response of immune complex or anti-dsDNA antibody. The LN patients treated with at least one of drugs (Pred, MMF and HCQ) were classed as drug treatment group (YES), otherwise they were classed as no-treatment group (NO). The difference between LN-Active and LN-Inactive were analyzed using Wilcoxon test, and the p values were indicated accordingly.

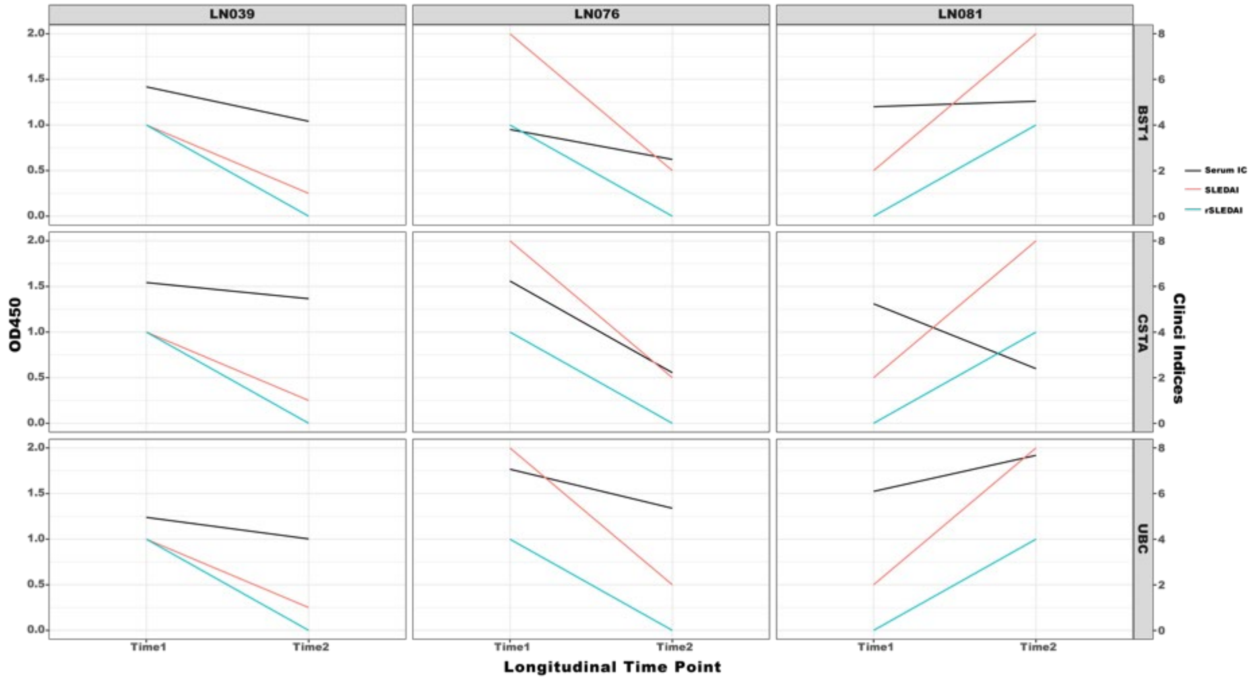

Supplementary Figure 3. Longitudinal changes in serum ICx level and disease activity in LN. Serum ICx levels (left, black) and clinic indices SLEDAI/rSLEDAI (right, red/blue) were serially monitored at two consecutive clinical visit in three LN patients. The interval between each visit was 6 months for patients LN039, LN076 and LN081.

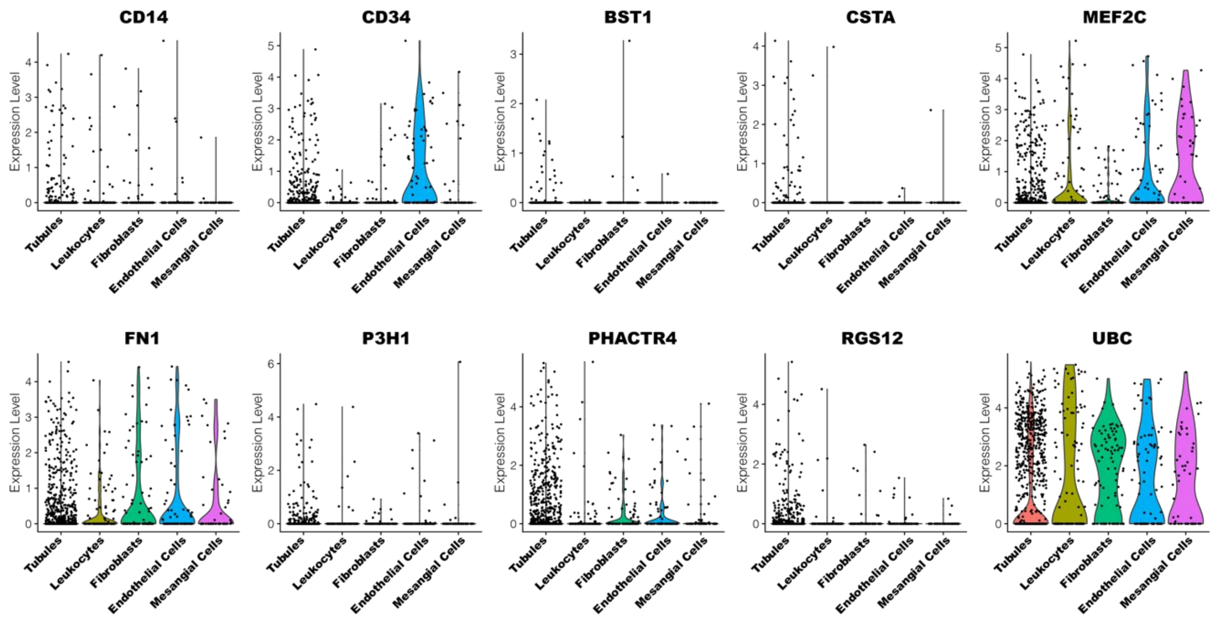

Supplementary Figure 4. Kidney scRNA expression of autoantigen candidates in LN. Violin plot analysis of kidney scRNA expression in 6 cell types from 1496 LN patients were performed using raw data from a scRNA seq database [1]..

REFERENCES

1. Der E, Suryawanshi H, Morozov P, Kustagi M, Goilav B, Ranabothu S, et al. Tubular cell and keratinocyte single-cell transcriptomics applied to lupus nephritis reveal type I IFN and fibrosis relevant pathways. Nature immunology. 2019; 20(7):915-927.
2. Nehar-Belaid D, Hong S, Marches R, Chen G, Bolisetty M, Baisch J, et al. Mapping systemic lupus erythematosus heterogeneity at the single-cell level. Nature immunology. 2020; 21(9):1094-1106.
3. Yao M, Gao C, Zhang C, Di X, Liang W, Sun W, et al. Identification of molecular markers associated with the pathophysiology and treatment of lupus nephritis based on integrated transcriptome analysis. Frontiers in Genetics. 2020; 11.
4. Buang N, Tapeng L, Gray V, Sardini A, Whilding C, Lightstone L, et al. Type I interferons affect the metabolic fitness of CD8+ T cells from patients with systemic lupus erythematosus. Nature communications. 2021; 12(1):1-15.
5. Zhu H, Mi W, Luo H, Chen T, Liu S, Raman I, et al. Whole-genome transcription and DNA methylation analysis of peripheral blood mononuclear cells identified aberrant gene regulation pathways in systemic lupus erythematosus. Arthritis research & therapy. 2016; 18(1):1-17.
6. Berthier CC, Bethunaickan R, Gonzalez-Rivera T, Nair V, Ramanujam M, Zhang W, et al. Cross-species transcriptional network analysis defines shared inflammatory responses in murine and human lupus nephritis. The Journal of Immunology. 2012; 189(2):988-1001.
